# Qualitative Evaluation of Water Displacement in Simulated Analytical Breaststroke Movements

**DOI:** 10.2478/v10078-012-0023-7

**Published:** 2012-05-30

**Authors:** Jonas Martens, Daniel Daly

**Affiliations:** 1 Department of Human Kinesiology, Faculty of Kinesiology and Rehabilitational Sciences, KULeuven, Leuven, Belgium.

**Keywords:** swimming, flow visualization, breaststroke

## Abstract

One purpose of evaluating a swimmer is to establish the individualized optimal technique. A swimmer’s particular body structure and the resulting movement pattern will cause the surrounding water to react in differing ways. Consequently, an assessment method based on flow visualization was developed complimentary to movement analysis and body structure quantification. A fluorescent dye was used to make the water displaced by the body visible on video. To examine the hypothesis on the propulsive mechanisms applied in breaststroke swimming, we analyzed the movements of the surrounding water during 4 analytical breaststroke movements using the flow visualization technique.

## Introduction

In swimming, a number of propulsion mechanisms have been proposed by Counsilman (1970), Colwin (1992), Maglisho (2003) and Persyn et al. (2003). These hypotheses were based on analogies with ship propulsion, i.e., a propeller-wing (known as lift) or a paddle (known as drag). Furthermore, some have suggested that the wave-like body movements sometimes seen in the breaststroke could also generate propulsion (McElroy and Blanksby, 1976; Persyn et al., s.d.). These mechanisms are comparable with those seen in some swimming animals (Persyn et al., 2003).

The aim of this study was to experimentally examine selected hypotheses by studying the displacement of water around a breaststroke swimmer using a flow visualization method with colored water.

### Propulsion and drag during limb movements in breaststroke swimming

#### Leg movement

In previous studies by Silva et al. (2003) and Verbrugge (2006), 62 elite breaststroke swimmers were investigated. The leg kick (from the first backwards leg movement to the end closing) begins at the slowest moment in the stroke cycle and caused the largest velocity increase of the center of mass (43.2 %) (Verbrugge, 2006) while representing only 28.52 % of the stroke cycle duration. In breaststroke the kick is responsible for a greater share of the total propulsion than in the other three competitive strokes.

During the kick, the propulsive surfaces must sweep laterally or vertically (relative to a fixed background) to move the body forward from the most ‘stable’ support in the water while slipping backward through the water as little as possible or even moving forward (Soons, 2003). The feet first move backward (paddling) and when fully extended, quickly close together (wing-like propulsion) (Maglisho, 2003).

This breaststroke kick causes a mass of water to accelerate in the wake behind the heels and feet (Soons, 2003). The first large velocity increase is due to the spreading (extension) of the legs with the feet pronated. During the first and last parts of the foot trajectory, the lift propulsion might actually be generated by the particular foot position and shape. The legs apply pure drag propulsion only during the middle part of the backward trajectory (Persyn et al., 2003). This motion is analogous to the propulsive system of a paddle wheel with movable blades, which also combines lift and drag propulsion (Van Lammeren, 1944).

The second, smaller velocity increase of the body’s center of mass is due to the first part of the closing action of the legs (from wide leg extension to the halfway closing of the legs). Here, the swimmer makes use of a propulsive mechanism analogous to a ship propeller. Because the velocity of the center of mass is high at the start of leg closing, the increase is smaller. During the first part of the leg squeezing, a horizontal propeller-like trajectory of the foot sole is seen. The velocity of the center of mass increases as the water is deflected backwards. Maglisho (2003) stated that to maximize propulsion during leg closing, the swimmer should turn the soles of the feet toward one another as they complete the leg extension (i.e., supination of the feet while squeezing). Ankle supination is easier and more effective in swimmers using body undulation in the breaststroke because they use a deeper kick. Keeping the soles of the feet vertical is easier during squeezing, when the legs are deeper and the angle of the ankle joint can be greater (Colman, 1991).

#### Arm movement

Silva et al. (2003) and Verbrugge (2006) also showed that the arm pull is responsible for a mean velocity increase of 26.6 %, representing the second largest increase in the breaststroke cycle. The arm movement accounts for 42.88 % of the cycle duration (Verbrugge, 2006). The total acceleration is smaller than that during the leg kick, possibly due to higher drag caused by the wide arm movement and the higher velocity at the start of this phase. The initial outward movement of the arms at the 100-m race pace that was studied actually decelerates the body (from 106.3 to 100 %) due to the disturbance of the streamlined position. Propulsion is generated during the spreading of the arms, i.e., by sculling, but at that moment, drag is still greater than propulsion. During this phase, the swimmer also changes body position from an optimal streamline during the glide to the least streamlined position during breathing.

Arrelano (2006) describes sculling as a basic propulsive movement characterized by an important application of hydrodynamic lift force. It is performed with an elliptical figure-8 trajectory of the hands in the horizontal plane. This movement is composed of 4 kinematic segments: two in translational phases (insweep and outsweep), when the hands move outward or inward through the water with an efficient angle of attack (approximately 40°), and two rotational phases (pronation and supination), when the hands rapidly rotate and reverse direction.

Various types of sculling movements are employed by swimmers, depending upon the actions and objectives, although the function of all sculling motion is to produce lift force through reciprocating motion. There is no consensus on the optimal hand configuration for sculling, so it is generally sought empirically by coaches and athletes. Schleihauf (1979) and Ito (2006) investigated the hydrodynamic characteristics of various hand configurations to determine which produced the maximum resultant force. Schleihauf found that the lift characteristics vary from sculling path to sculling path and that the maximum resultant force increased up to an angle of pitch of approximately 40° and then decreased. To generate maximum propulsion, the fingers should be closed and the thumb fully abducted. Ito agreed and added that a cupped hand with straight fingers is better than naturally bent fingers.

When the hands are pulled past shoulder level after spreading and before the squeezing of the arms, a long, large thrust is generated if the forearms are positioned properly. This long arm motion is, however, detrimental in breaststroke swimmers using a flat style (no body undulation). Greater thrust is generated, but drag is also increased because of the longer hand recovery. Breaststroke swimmers with an undulating style (with body waving and trunk camber) benefit from a longer pull (Verbrugge, 2006), and they are hindered less by drag during recovery, probably because they are able to bring their arms forward above the water surface. The undulating style swimmers examined by Verbrugge (2006) all had a high arm recovery in contrast to those studied by Vilas-Boas (1996), who distinguished between swimmers with an “overwater” and an “underwater” recovery.

Verbrugge (2006) pointed out that undulating breaststroke swimmers also benefit from a longer hand path. They spread the arms not only sideward but upward and then bring the arms together deep in the water. With a longer path, a larger water mass is accelerated backwards. The deep arm squeezing also enables the swimmer to lift the trunk higher out of the water for breathing, permitting higher arm recovery.

### Propulsion and drag during trunk movements in breaststroke swimming

#### Trunk rotations (above the water surface)

During the first part of the arm pull, the undulating breaststroke swimmer accelerates more than the flat breaststroke swimmer. The undulating swimmer uses a part of the energy to lift the trunk out of the water (Persyn and Colman, 1999) by deflecting water downwards. In contrast, the flat swimmer probably uses all his energy to deflect water backwards. The swimmer using only trunk cambering without undulation achieves trunk rotation by deflecting water downward due to the upward leg kick and by contracting the back muscles. This cambering action results in less deceleration during recovery compared with a flat style swimmer (Persyn and Colman, 1999). This observation can be explained by the following:

1. Lower drag because of the above-water arm recovery,
2. Body segments (e.g., upper trunk, shoulder girdle, arms and head) are accelerated forward above the water surface. At the same time, the body parts below the surface are displaced backward. When combined with kicking, more “grip” is obtained on the water (less slip). Because of the greater density of water compared with air, the rearward displacement of segments under water is shorter than the forward displacement above water. Consequently, the total body center of mass moves forward (Persyn et al., 2003).
3. The inertia of the added mass of water that is set in motion behind an accelerating body segment (trunk) can push the body forward when this body segment decelerates during the following phase (breathing).

The results above are based on cross-sectional statistical analyses. These results nevertheless need further experimental confirmation. To examine these proposed concepts, the series of four experiments presented in Table 1 were performed.

## Material and methods

### Participants

A total of eleven trained swimmers (nine male, two female) performed various analytical breaststroke-like movements. One participant was an international-level freestyle swimmer, seven were national-level breaststroke swimmers in their age category, and three were movement science students specializing in swimming. All participants agreed to take part but were kept naïve to the intention of the experiments.

### Video Measurements

To visualize the water flow around the hand, foot sole or head during breaststroke swimming, a tape, made of a wad of cotton wool and containing 1 gram sodium fluorescelnate powder (Riedel-de Haën), was attached on specified positions on the body of the swimmer with Hansaplast tape. An overview of the experiments with their respective tape positions is given in Table 1. The swimmer took a position in the water before each experiment with the body part to be examined held above the surface. The skin at the appropriate tape position was first dried, and then the tape was applied. The swimmer then carried out the required movement. When this tape was submerged, the dye dissolved and colored the water displaced by the arm and leg movements.

When attached to the hand or foot sole, the limb that was closest to the camera was chosen for visibility reasons. We assumed symmetrical movements as this is required by the breaststroke competition rules.

The various experiments were recorded on video tape with a 25-Hz camera (Sony-DV) from an underwater side view. The camera was placed behind an underwater window perpendicular to the swimming direction. The video-sequence of each experimental condition was transferred to PC using Adobe Premiere software. The movement of the water around the swimmer was then analyzed in the following manner. A photo series at intervals of 0.08 s was made of the entire experimental condition, starting from the first body movement with the attached dye and continuing until after the dye was completely mixed with the water after a particular movement ended. This interval was selected to gain sufficient information while keeping the computer processing and analysis time reasonable (normal video images consist of 25 frames/second, which is 0.04 seconds between the images -by taking only half of the images, the process speed is doubled ). Filming and analyzing four experiments for 1 swimmer took approximately 4 hours. After observation, the most relevant photos were selected, and a time code was noted using the timer of the Adobe Premiere video player. The clouds of dye were located and outlined on the selected photos using Microsoft Paint by repeatedly playing the video sequence forward and backward. Together with the cloud outline, reference lines were added to the photos to allow comparison of the cloud locations from one instant to another.

### Experimental Procedures

The 11 swimmers each performed analytical simulations of 4 breaststroke movements or parts of movements with the dye after practicing each simulation without dye until the skill was mastered.

#### Experiment 1: Ankle Supination

The swimmer was asked to perform spreading and squeezing movements with the legs while lying on the back in the water. First, the participant executed these movements without supinating the feet during leg squeezing, followed by supinating the feet. The participants performed this simulation while holding a kickboard on the stomach to prevent any sculling movements of the hands and to stay afloat more easily. This experiment was started from a still position in the water.

#### Experiment 2: Sculling

Lying on the stomach, the subject had to scull with the hands with the elbows just below water level and without pushing off of the wall. The elbows had to be at shoulder level with a 90° angle between the forearm and upper arm. There was no flexion or extension allowed in the wrist joint as the hand and forearm had to be in one line. This position was controlled by observing (video) the sculling action from the underwater side view.

#### Experiment 3: Paddling

The swimmer lay on the stomach and performed one backward paddling movement with both arms as close to the water surface as possible without breaking the surface, as if a rowing boat with two paddles (his arms). The swimmer was not allowed to push off the wall.

#### Experiment 4: Added mass on back

The participant was asked to strongly push off the wall with the hands forward and then rotate the trunk explosively upward and hold his body still at this moment with the hands in the breaststroke recovery position (90° angle in the elbows).

Some participants executed some experiments more than once with dye. In those cases, the trial executed best was chosen for analysis. The average duration of filming one subject was one hour.

## Results

The various trials from all swimmers were analyzed and compared. Only the trials performed correctly will be presented here.

### Limb movements

#### Leg movement

##### Experiment 1: squeezing the legs with or without supination

**Figure 1:**
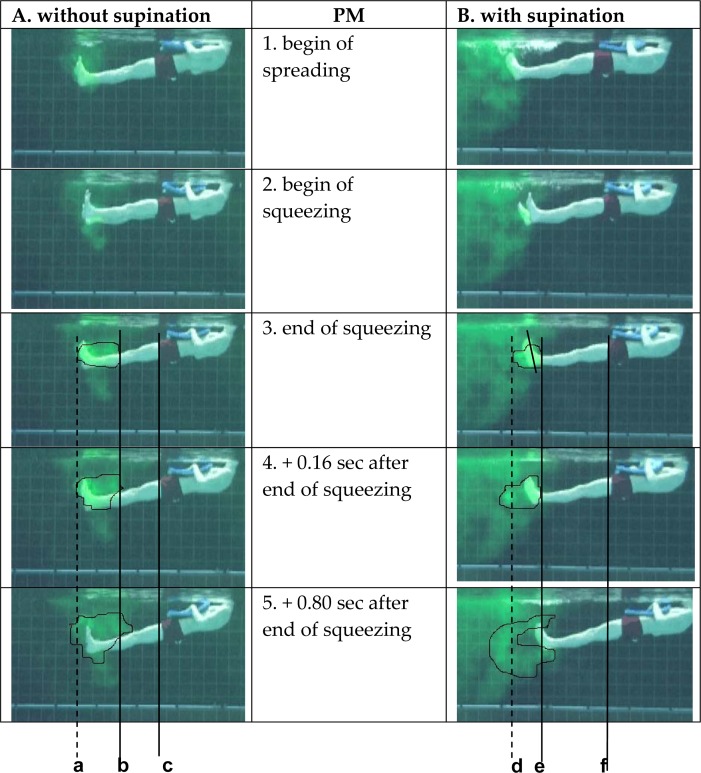
Column A shows the squeezing action when PM does not supinate the feet. In column B, PM supinates the feet. Reference lines a, b, d and e were added at the end of the squeezing phase to analyze the movement of the colored water mass. The dotted lines, a and d, indicate the furthest part of the cloud, and lines b and e indicate the nearest part of the cloud. Reference lines c and f were added to analyze the forward movement of the swimmer and are related to the waistband of the suit.

Figure 1, column A shows a leg closing action with dorsi flexion but without ankle supination. Almost no backward movement (to the left) of the colored cloud (water) is seen (lines a & b). Some water even moves forward (to the right) (line b). The cloud becomes larger (photo 3 versus photo 5). The swimmer hardly moves to the right (line c). In column B, the participant supinates the feet. The cloud (water) is accelerated backward (lines d & e), and following the law of action-reaction, the swimmer moves forward, as seen in photo 5, taken at + 0.80 seconds after the end of the squeezing action.

#### Arm movement

##### Experiment 2: sculling

**Figure 2:**
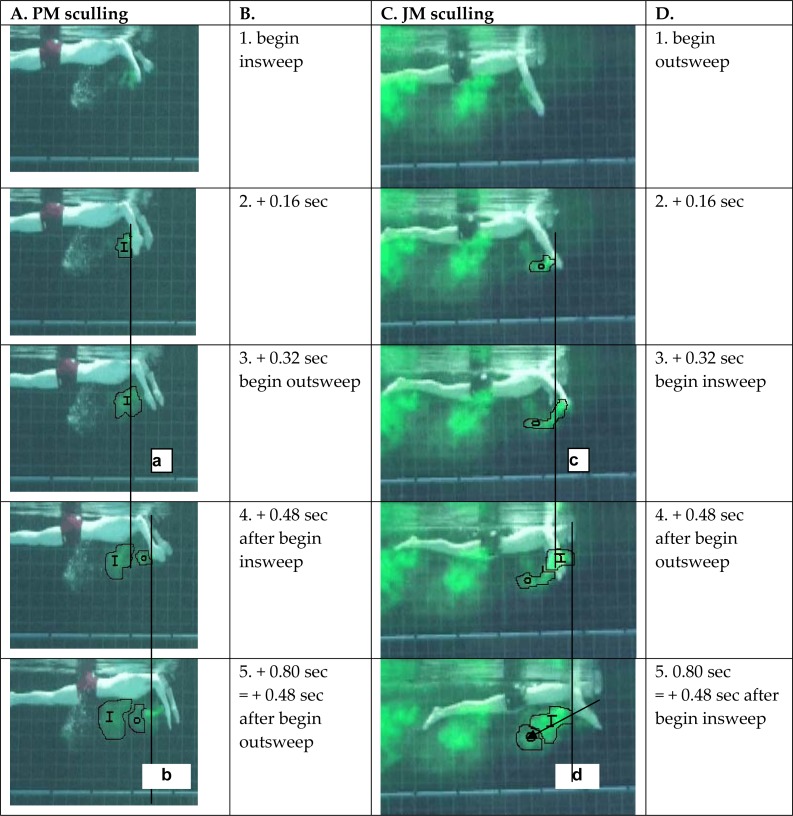
In column A, the sculling action of PM is depicted, and in column C, the sculling action of JM is depicted. Time codes have been added in columns B and D. Reference lines a and b were added to analyze the movement of the colored water mass, with a referring to the insweep (I) and b to the outsweep (O) of PM. Reference line c refers to the outsweep (O), and d refers to the insweep (I) of JM. We compared the movement of the clouds using the reference lines at + 0.48 sec after the beginning of the sweep.

Two sculling experiments are shown in Figure 2. In both swimmers, the inward sculling sweep (lines b & d) creates a larger cloud than the outward hand sweep, but in 1 swimmer (column C), the inward cloud receives a larger acceleration backward when comparing the photos taken at + 0.48 seconds after the sweep (photos 4 & 5). In column A of Figure 2, the inward sweep also created a larger cloud, but here the outward sweep produced a larger acceleration of water when comparing the photos taken at + 0.48 seconds after the sweep is given (photos 4 & 5 for column A).

One of the two participants accelerated the water purely backwards (Column A). The swimmer in column C directed the clouds slightly downward. The angle of the forearms relative to the vertical reference lines was also larger in JM (Column C) than in PM (Column A).

##### Experiment 3: paddling

**Figure 3:**
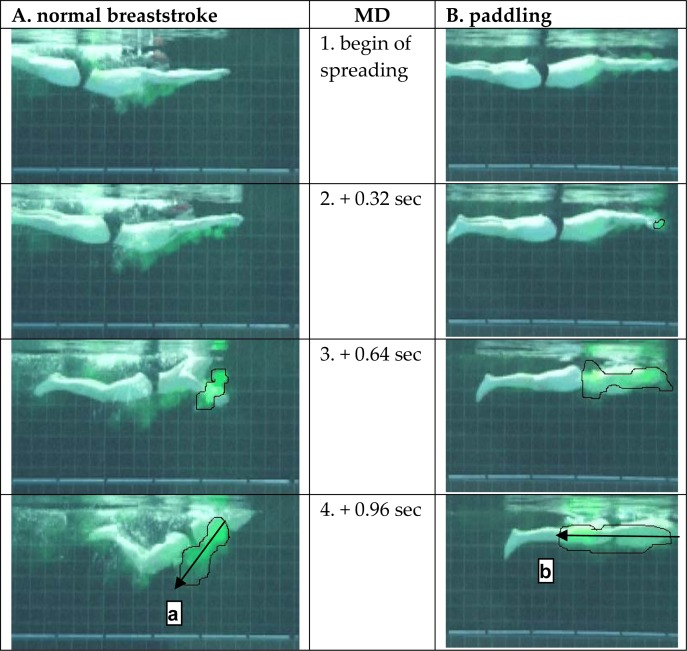
In column A, the normal breaststroke of MD is depicted, and in column B, the performance of the analytical paddle movement is depicted. Arrows a and b were added to indicate the direction of the colored mass of water.

A purely backward water movement can be seen by following arrow b in Figure 3, which indicates the direction of the colored mass of water. In the normal breaststroke arm action, however, a similar direct rearward displacement of colored water is not seen (arrow a). The direction of the cloud is backward-downward.

### Body movements

#### Trunk rotations

##### Experiment 4: Added mass pushed in back

**Figure 4:**
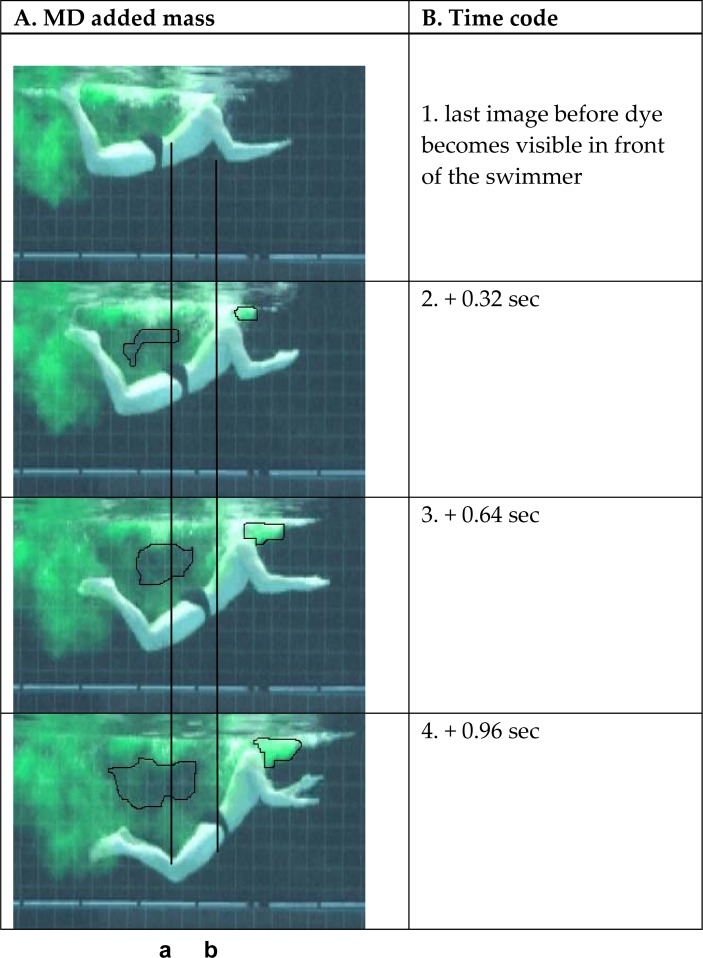
In column A, the experiment with the added mass that is pushed in the back, as performed by MD, is depicted. A time code was added in column B. Reference line a was added to analyze the forward movement of the swimmer, indicating the waistband of the suit in photo 2. Reference line b was added to analyze the movement of the colored water mass. An area behind the swimmer where the dye disappears is also indicated.

All the successful simulations showed the same flow pattern. The clearest example is given in Figure 4.

The amount of colored water increases in front of the swimmer (Figure 4, line b), and the waistband of the swimmer’s suit moves forward over time (Figure 4, line a). Behind the swimmer, the mass of non-dyed water is visible and increases in size.

## Discussion

In this study, a flow visualization technique using colored water was applied to evaluate a qualitative breaststroke technique in eleven participants. The eleven swimmers performed various analytical breaststroke-like movements, requiring them to feel the difference between the effects of specific swimming movements and the effects on their bodies. To visualize the water movement caused by the arm and leg movements, a cotton ball containing sodium fluorescelnate powder was taped to the propelling body surface. Unsuccessful experiments during which insufficient dye was dissolved or the tape was loosened occurred mostly during the trials of the first participant, a movement science student who was not a competitive swimmer. In the following participants, the experience of the researchers prevented such technical failures most of the time.

### Limb movements

#### Leg movement

##### Experiment 1: squeezing the legs with or without ankle supination

This movement variation was studied to examine the effect of supinating the feet compared with not supinating. All swimmers readily agreed concerning the importance of supinating the feet in attaining speed both during the experiment itself and after watching the video sequences. There was little or no forward body movement during leg squeezing if the feet were not supinated. When the swimmer supinated the feet, the water was displaced backwards, and following the law of action-reaction (Figure 1), the swimmer moved in the opposite direction (Counsilman, 1968; Councilman, 1970; Dickinson, 2000). This result further supports the importance of ankle supination in the breaststroke, as was previously pointed out by Maglisho (2003).

#### Arm movement

##### Experiment 2: sculling

All participants were familiar with sculling because it is a very popular exercise in swimming workouts. The goal of sculling exercises is to attain a better “feeling” of the water, permitting the swimmer to find the optimal hand position to produce propulsion.

The inward sculling sweep created a larger cloud of moving water than the outward sweep. In 1 swimmer, the cloud was accelerated backward. The outward sweep also deflects water backwards, but only if the hand is positioned correctly (Schleihauf, 1979; Arrelano, 2006; Ito, 2006). In swimmers, the internal rotators and flexors of the arm musculature are more developed than the external rotators and extensors. Moreover, in the freestyle, breaststroke and butterfly, an inward sweep is seen in the hand movement when the arms are at shoulder level, which is the position required in this experiment. Thus, this observation might further provide evidence that a swimmer’s musculature is more developed for inward sculling movements than for outward sculling movements.

Interestingly, one of the two participants essentially directed the acceleration of the water backwards, and therefore all energy was used to move forward (Figure 2, column A). This movement was achieved by positioning the forearms and hands perpendicular to the water surface. In Figure 2, column C, however, the participant directed the water more bottomward by positioning the forearms and hands at an angle relative to the vertical reference line. The swimmer received feedback on the observation that he lost energy by this arm position, which might have been done to keep the head or shoulders higher relative to the water surface.

##### Experiment 3: paddling

The basic concept of this experiment was to let the swimmers feel that it is not easy to paddle with the hands without sculling. These 2 movements are often combined in swimming.

The comparison of an analytical paddle movement with a normal breaststroke pull is shown in Figure 3. The paddle movement can easily be compared to a rowboat using 2 paddles. A purely backward water displacement can be seen as a consequence of this rowing action. In the normal breaststroke, however, a straight backward displacement of water is not seen and was not expected. The swimmer rotates the trunk to breathe, so some water has to be deflected downward. This basic form is what Persyn and Colman (1999) described as one of the differences between a flat breaststroke swimmer and an undulating breaststroke swimmer. The undulating swimmer uses a part of his energy to climb high out of the water, allowing an above-water arm recovery, whereas the flat swimmer uses this energy to paddle forward.

### Body movements

#### Trunk rotations

##### Experiment 4: Added mass of water pushing on the swimmers back

The analytical simulation in which the swimmers pushed off the wall followed by an explosive upward trunk rotation was meant to precisely examine the effect of this explosive movement. All four swimmers felt something they described as a “backpack” of water being pushed into their necks. They were not surprised when they saw the colored cloud of water moving around their necks on the video sequences.

Figure 4 shows that the inertia of the added mass of water that closes in behind an accelerating body section can maintain the forward movement of that body section, as described by Persyn and Colman (1999) and Persyn et al. (2003). Because the swimmer is moving with sufficient velocity when he rotates the trunk upward, a mass of water is pushed against his decelerating body from behind the back. Behind the swimmer, a mass of non-dyed water is visible and grows. The water that pushes the swimmer forward disappears here, becoming visible in front of the swimmer, and uncolored water fills the space that the colored water previously occupied.

## Conclusions and recommendations

In this study, a qualitative method to evaluate swimmers based on a water flow visualization technique is presented, complimentary to traditional movement analysis. The visualization technique used follows the water flow and focuses on the action-reaction principle. The dye particles have neutral buoyancy, whereas, for example, air bubbles rise to the water surface on their own, making the analysis of displaced water difficult. Moreover, the technique did not hinder the swimming movement or the water and is low cost. Finally, this technique enabled quick preparation of the swimmer for performing a movement simulation. The main disadvantage was the relatively slow clearance of the dye in the water. This issue was substantially reduced by actively pushing the colored water towards the filters.

In our experiments, the tape containing the powder loosened more frequently when attached to the foot sole. The problem was solved by ensuring that the body surface was completely dry before attaching the tape. In the future, each swimmer should perform all movement variations several times. This repetition was not done systematically in this study due to the limited availability of some participants and technical problems. Furthermore, the problem of dye clearance limited the number of trials possible within a time period.

Studying the effects on learning by providing feedback based on the images of their own movements using the visualization method could also be interesting. This study could include a series of evaluations in which one group of subjects does not get the visual feedback of the video sequences accompanied by directions on technique from an expert and the other group does get the video feedback and the directions on technique from an expert. Providing swimmers with a greater insight into their movements can improve their performances, as already described in the study of Colman et al. (2006) involving one freestyle swimmer.

## Figures and Tables

**Table 1:** Overview of the experiments with their respective tape positions

	Experiment Analytical simulations	Position of the tape
Experiment 1	Supination	Left foot sole
Experiment 2	Sculling	Right hand palm
Experiment 3	Paddling	Right hand palm
Experiment 4	Added mass on back	Back of the head and between shoulders
